# The Local Damage and Systemic Inflammation Induced by a Biodegradable Polydioxanone Stent Implanted in the Rabbit Trachea Decreases Markedly with Stent Degradation

**DOI:** 10.3390/ijms27031309

**Published:** 2026-01-28

**Authors:** Carolina Serrano-Casorran, Sergio Rodriguez-Zapater, Francisco Rodriguez-Panadero, Raquel Gomez, Cristina Bonastre, Jose Andres Guirola, Jose Rodriguez, Miguel Angel de Gregorio

**Affiliations:** 1 Minimally Invasive Research Group (GITMI), Universidad de Zaragoza, 50009 Zaragoza, Spain; carolina.serrano@unizar.es (C.S.-C.); cbonastr@unizar.es (C.B.); jaguirola@unizar.es (J.A.G.); jrodgom@unizar.es (J.R.); mgregori@unizar.es (M.A.d.G.); 2 Department of Animal Pathology, Universidad de Zaragoza, 50013 Zaragoza, Spain; 3 Department of Animal Sciences, Universitat de Lleida, 25198 Lleida, Spain; 4 Instituto de Biomedicina de Sevilla (IbiS), Hospital Universitario Virgen del Rocío, CSIC-Universidad de Sevilla, 41013 Sevilla, Spain; frodriguezpan@gmail.com (F.R.-P.); rgomez-ibis@us.es (R.G.); 5 Unidad Medico-Quirurgica de Enfermedades Respiratorias (UMQER), Hospital Universitario Virgen del Rocío, 41013 Sevilla, Spain; 6 Department of Microbiology, Pediatrics, Radiology and Public Health, Universidad de Zaragoza, 50009 Zaragoza, Spain; 7 Interventional Radiology Department, Lozano Blesa University Hospital, 50009 Zaragoza, Spain; 8 Interventional Radiology, Clínica Quirón, 50012 Zaragoza, Spain

**Keywords:** stents, absorbable implants, polydioxanone, tracheal stenosis, cytokines, interleukin-8, models, animal

## Abstract

Biodegradable tracheal stents have been developed to overcome the limitations of metallic and removable stents in benign airway disease. This study evaluated the local and systemic inflammatory response induced by a biodegradable polydioxanone tracheal stent in a rabbit model. Twenty-one rabbits were assigned to three follow-up groups (30, 60, and 90 days). In each group, six animals received a tracheal stent, and one served as a sham control. Clinical status and respiratory symptoms were monitored, and serial peripheral blood interleukin-8 (IL-8) levels were measured. At the end of follow-up, tracheoscopy, IL-8 quantification in tracheal lavage, and necropsy were performed. No deaths or severe respiratory symptoms occurred. Tracheoscopic findings were significantly less severe after stent degradation, with reduced congestion (*p* = 0.030), inflammation (*p* = 0.003), and secretions (*p* = 0.030). Two granulomas and two cases of stenosis were identified. Mean IL-8 expression in tracheal lavage (relative quantification, RQ) was 14,129 ± 3007 when the stent was present and 426 ± 100 after degradation (*p* = 0.003). Blood IL-8 expression increased significantly on day 1 compared with baseline (*p* = 0.022) and subsequently decreased (*p* = 0.034). Inflammatory and structural alterations induced by a polydioxanone tracheal stent decrease after stent degradation.

## 1. Introduction

Tracheobronchial stents can provide rapid and effective relief of airway narrowing [1,2]. However, their use is associated with relevant limitations. Despite their initial clinical benefits, self-expandable metallic stents are contraindicated in benign airway disease because of long-term complications and the difficulty, or even impossibility, of removal [3,4,5]. To address these limitations, biodegradable stents have been developed. These devices are designed to maintain airway patency for a defined period and then progressively degrade and disappear.

Polydioxanone is a biodegradable polymer with a relatively long degradation time, making it suitable for the manufacture of airway stents. Polydioxanone stents have been evaluated in several preclinical and clinical studies [6,7,8,9,10,11]. These stents degrade through hydrolysis and, because they are not permanent and do not require removal, they are expected to cause fewer long-term complications than conventional metallic or silicone stents.

Restenosis is the most frequent complication after airway stenting [12], and inflammatory processes play a central role in its development [13]. Tracheal intubation during surgical procedures lasting a median of three hours has been shown to significantly increase cytokine levels [14]. Early assessment of the inflammatory response by measuring pro-inflammatory and pro-fibrotic cytokines may help predict the development of tracheal stenosis after stent implantation. In a rabbit model, Arellano-Orden et al. demonstrated that interleukin-8 (IL-8) expression predicts the development of tracheal stenosis following metallic stent implantation [15].

However, despite the increasing use of biodegradable tracheal stents, the local and systemic response associated with their implantation and degradation remains poorly characterized. Therefore, the present study evaluated the tracheal and systemic responses to implantation of a biodegradable polydioxanone tracheal stent in a rabbit model. Clinical evolution, tracheoscopic findings, and IL-8 expression in blood and tracheal lavage were analyzed in order to characterize both local and systemic inflammatory responses to the stent and to assess their evolution during stent degradation.

## 2. Results

### 2.1. Clinical Follow-Up

**Figure 1:**
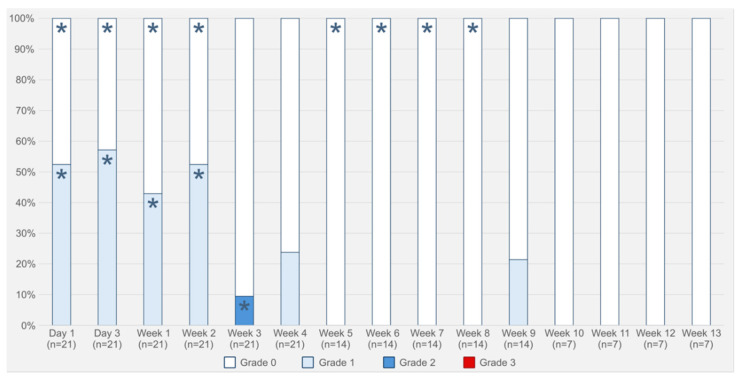
Evolution of the respiratory symptoms in rabbits with stent implanted. Grade 0: no respiratory symptoms; Grade 1: minor respiratory symptoms only under stress conditions (when manipulated); Grade 2: minor respiratory symptoms at rest; Grade 3: severe respiratory symptoms. *p* < 0.001 (likelihood-ratio test). * Observed value significantly lower or higher (*p* < 0.05) depending on the adjusted standardized residuals.

No animals died during the procedures or the follow-up period. No severe respiratory symptoms were observed. The evolution of respiratory symptoms over time is shown in Figure 1.

### 2.2. Tracheoscopic Findings

Tracheoscopic findings are summarized in Table 1 and Table 2. The stent was visible in all animals euthanized at 30 days, in 50% of animals at 60 days, and in none of the animals at 90 days. When present, the stent was intact and had lost its violet coloration. When the stent was no longer visible, no residual fragments were detected.

No significant differences in congestion or inflammation were observed among the three follow-up groups. However, significant differences were detected when cases were analyzed according to stent degradation status (*p* = 0.030 for congestion and *p* = 0.003 for inflammation). Severe congestion occurred more frequently when the stent was still present, whereas no inflammation was detected in any animal after complete stent degradation.

Secretion accumulation differed significantly among follow-up groups (*p* = 0.007) and according to degradation status (*p* = 0.030). Moderate or abundant secretions were always observed when the stent was present. After stent degradation, only moderate or absent secretions were detected.

**Figure 2:**
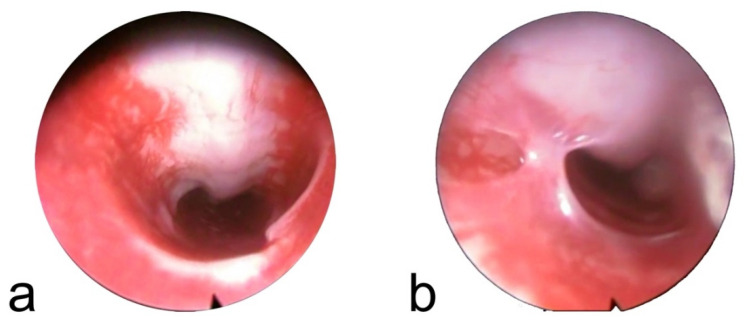
Tracheoscopy images compatible with granuloma. (**a**) Tissue growth in the ventral area of the trachea in an animal with 90 days of follow-up and the stent degraded. (**b**) Tissue strip that connected the ventral and dorsal zones of the trachea in an animal with 60 days of follow-up and the stent degraded.

Endoscopic images compatible with granulomas were identified in two animals: one in the 60-day group and one in the 90-day group, in both cases after stent degradation (Figure 2). In both animals, the lesions consisted of tissue overgrowth located in the cranial trachea. One lesion was located on the ventral tracheal wall, and the other consisted of a tissue bridge connecting the ventral and dorsal walls.

Tracheal stenosis was observed in two animals. In the 30-day group, stenosis was associated with severe inflammation in the presence of the stent. The second case of stenosis corresponded to the granulomatous tissue bridge observed in one animal from the 60-day group after stent degradation.

### 2.3. Inflammatory Response: IL-8 Expression

**Figure 3:**
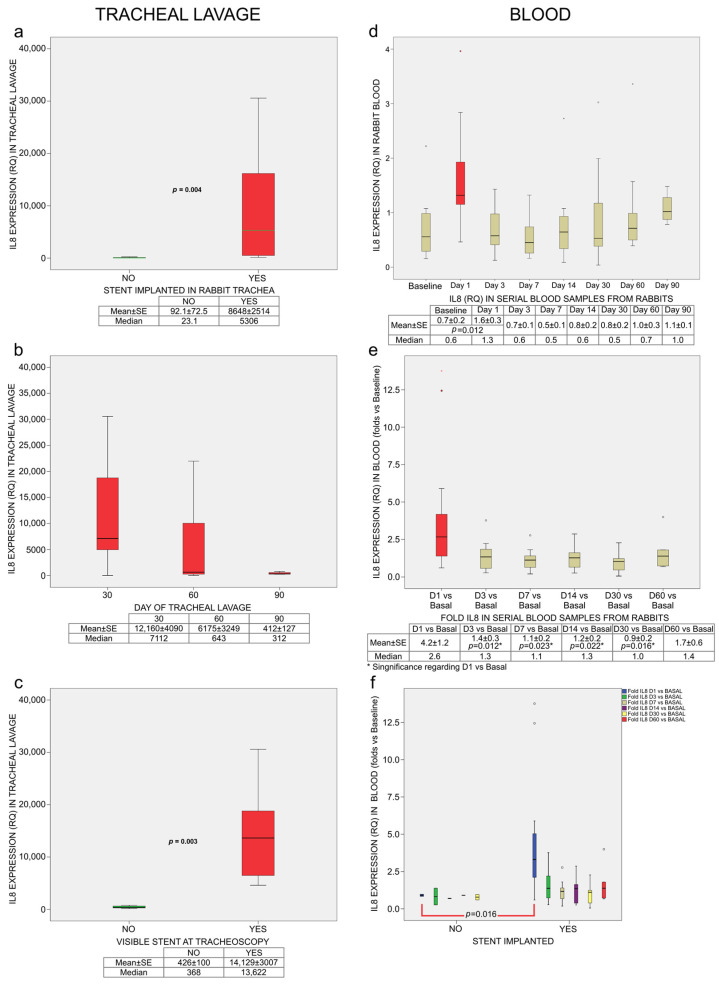
IL-8 Expression in tracheal lavage and blood (RQ, Relative Quantification). (**a**) IL-8 expression in tracheal lavage (sham control vs. implanted stent). (**b**) IL-8 expression in tracheal lavage (euthanasia on 30, 60, and 90 post-implantation day). (**c**) IL-8 expression in tracheal lavage (visibility of the stent in the tracheoscopy). (**d**) IL-8 expression in serial blood samples (baseline and days 1, 3, 7, 14, 30, 60, and 90 post-implantation). (**e**) IL-8 expression in serial blood samples (Folds IL-8 expression [RQ] on days 1, 3, 7, 14, 30, and 60 post-implantation vs. baseline). (**f**) IL-8 expression in serial.

IL-8 expression in tracheal lavage was significantly higher in stented animals (Relative Quantification [RQ]: 8648 ± 2514) than in sham controls (RQ: 92.1 ± 72.5; *p* = 0.004) (Figure 3a). At 30 days, mean IL-8 expression was 12,160 ± 4090; at 60 days, 6175 ± 3249; and at 90 days, 412 ± 127 (*p* = 0.191) (Figure 3b).

**Figure 4:**
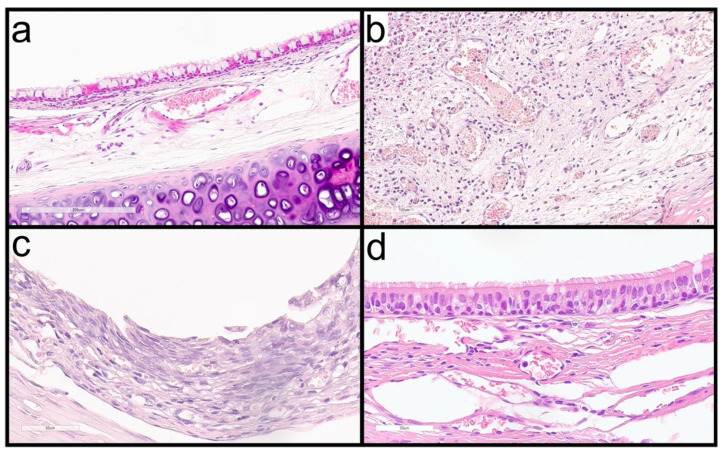
Histological images. (**a**) Example of moderate epithelial changes in an animal with 60 days of follow-up and the stent degraded. (**b**) Example of neovascularization (severe changes quantity) in an animal with 30 days of follow-up and no degraded stent. (**c**) Example of scamous metaplasia in an animal with no degraded stent. (**d**) Example of a mild alteration epithelium in an animal with 90 days of follow-up and the stent degraded.

IL-8 expression strongly correlated with stent visibility on tracheoscopy (Spearman’s ρ = 0.850, *p* < 0.001). When the stent was present, IL-8 levels were 14,129 ± 3007, whereas after stent degradation they decreased to 426 ± 100 (*p* = 0.003) (Figure 3c). IL-8 expression in tracheal lavage also correlated with the presence of squamous metaplasia (ρ = 0.779, *p* < 0.001) and with the degree of neovascularization observed in histological analysis (ρ = 0.786, *p* = 0.002) (Figure 4).

Baseline blood IL-8 expression was 0.7 ± 0.15 RQ. On day 1 post-implantation, IL-8 levels increased to 1.6 ± 0.3, which was significantly higher than baseline (*p* = 0.022) (Figure 3d). Fold changes relative to baseline on day 1 differed significantly from those on days 3 (*p* = 0.034), 7 (*p* = 0.023), 14 (*p* = 0.022), and 30 (*p* = 0.016) (Figure 3e). On day 1, IL-8 fold changes were also significantly higher in implanted animals than in sham controls (*p* = 0.016) (Figure 3f).

Blood IL-8 fold changes on day 1 correlated with the degree of epithelial alteration in histological samples (ρ = 0.574, *p* = 0.004), as did IL-8 expression on day 3 (ρ = 0.721, *p* = 0.008) (Figure 4). IL-8 fold changes on day 7 correlated with respiratory symptoms observed during the second week (ρ = 0.726, *p* = 0.011), and IL-8 expression at 30 days correlated with symptoms observed during the fourth week (ρ = 0.655, *p* = 0.003).

## 3. Discussion

Self-expandable metallic stents have several properties that make them attractive for the treatment of tracheobronchial stenosis. They can be implanted using flexible bronchoscopy or under fluoroscopic guidance, have a thin wall profile that maximizes luminal diameter, and allow mucociliary clearance and re-epithelialization through their mesh structure within a few weeks [3,5,16,17,18]. However, metallic stents are associated with severe complications, including granulation tissue formation, restenosis, migration, stent fracture, secretion retention, halitosis, hemoptysis, infection, and tracheoesophageal fistula. Furthermore, although rapid re-epithelialization may initially appear advantageous, it complicates or even prevents stent removal [3,4,5,16]. For these reasons, the United States Food and Drug Administration (FDA) has issued a warning against the use of metallic stents in benign tracheobronchial disease [19].

Silicone stents are currently considered the gold standard for benign airway stenosis because they are relatively safe, inexpensive, well tolerated, and removable [3,5,16,18,20,21]. However, their placement requires rigid bronchoscopy, and complications such as mucus plugging, migration (particularly in stenotic airways), and granulation tissue formation at the stent ends remain common [3,18,21,22,23].

Following the FDA warning, covered metallic stents were developed in an attempt to combine the ease of deployment of metallic stents with the removability of silicone stents. Nevertheless, these devices also share disadvantages from both categories, including migration, granulation tissue formation, infection, and stent fracture [3,24].

An alternative strategy to reduce long-term complications is the use of biodegradable materials. Biodegradable stents, composed of degradable polymers or corrodible metals, are designed to provide temporary mechanical support and then progressively degrade after fulfilling their function [25].

Polydioxanone is among the most widely used biodegradable polymers for tracheobronchial stents [26]. Polydioxanone stents have been investigated in both non-pathological and pathological animal models, as well as in adult and pediatric clinical series [6,7,8,9,10,11].

The rabbit is a widely used animal model in airway stent studies [6,7,8,9,15]. Rabbit tracheal anatomy and epithelial repair mechanisms closely resemble those of humans, particularly with respect to mucociliary clearance and fibro-inflammatory responses, supporting the translational relevance of this model for airway stent evaluation.

In the present study, all animals survived the predefined follow-up period without developing severe respiratory symptoms. These results contrast sharply with those of a previous rabbit study performed by our group using self-expandable metallic stents, in which mortality rates reached 80% with stainless steel stents, 20% with nitinol stents, and 40% with paclitaxel-eluting nitinol stents [27]. Our findings are consistent with previous reports describing low clinical impact of polydioxanone stents in animal models [6,7]. Morante-Valverde et al. observed progressive stridor and reduced food intake due to obstructive granulation tissue in 19% of animals; however, in most cases stenosis developed only after repeated stent implantations [7].

Tracheoscopic evaluation revealed significant differences between animals in which the stent was still present and those in which it had already degraded. In contrast, few differences were observed between predefined follow-up time points, with the exception of secretion accumulation. Congestion was less severe after stent degradation, although some degree of congestion was still detected. Notably, two of the three sham control animals also exhibited severe congestion, suggesting that external or procedural factors may have influenced these findings and that stent-related congestion may have been overestimated.

Inflammation was detected only in animals in which the stent was still present and was generally mild. Only one case of severe inflammation was observed, which was associated with tracheal stenosis. Necropsy findings in this animal confirmed marked tracheal wall inflammation, consistent with previous observations by Morante-Valverde et al. [7].

Sham control animals showed no secretion accumulation, whereas animals with a stent consistently exhibited moderate or abundant secretions. After stent degradation, secretions were moderate or absent and did not cause airway obstruction. The radiopaque markers of the stent were the sites where secretions tended to accumulate most. We hypothesize that the relatively large filament diameter and the presence of radiopaque markers promote mucus retention and therefore warrant close clinical monitoring.

Tissue overgrowth compatible with granuloma formation was observed in two animals, both after stent degradation. The cranial and ventral localization of both granulomas, coinciding with the tracheostomy site and not with the stented segment, suggests a procedural rather than an implant-related origin. Granulation tissue is one of the most common complications of airway stenting in both experimental and clinical settings [4,5,10,12,16,18,20,21,23]. In animal models using polydioxanone stents, granulomas have mainly been reported in pathological airways [8,9] or after repeated stent placements [7]. In clinical series, granulation tissue has been the most frequent complication, with reported rates of 23.1% in adults and 34.6% in pediatric patients [10,11]. In contrast, metallic stents induce extensive granulation tissue formation even in healthy airways, making them useful as negative controls in experimental studies [27,28,29,30]. These findings contrast with the limited granulation tissue growth observed in the non-pathological model used in the present study and in the study by Choi et al. [9]. The granulation tissue formation reported with polydioxanone stents may be related to interactions between the stent and pre-existing lesions or to chronic stimulation associated with repeated stenting, rather than to the stent material itself, as has been described for metallic stents.

IL-8 was selected as a biomarker of stent reactivity because it is an early predictor of stent-induced tracheal stenosis. In contrast, other cytokines, such as bFGF, TGF-β, and VEGF, were found to be less informative than IL-8 expression in a previous study conducted by our group [15].

In humans, IL-8 is produced by airway epithelial cells in response to environmental stimuli and plays a role in neutrophil recruitment. Excessive recruitment can cause organ damage due to neutrophil infiltration and chronic inflammation [31,32]. Additionally, IL-8 is chemotactic for fibroblasts, accelerates their migration, and stimulates the deposition of tenascin, fibronectin, and collagen-I during in vivo wound healing [33,34]. Metallic stents increase IL-8 expression in in vitro human respiratory fibroblasts and in in vivo rabbit models, and this marker has been used in comparative studies on tracheal stents [12,15,28,35].

In this study, IL-8 levels in tracheal lavage were highest at 30 days and progressively decreased at 60 and 90 days. Although differences among time groups were not statistically significant, IL-8 levels differed markedly according to stent presence, with a strong positive correlation (72.3%) between IL-8 expression and stent visibility. Animals with higher IL-8 levels also showed more pronounced histological changes, including neovascularization and squamous metaplasia. The near-absence of clinically relevant respiratory symptoms despite high local IL-8 expression highlights the favorable tolerance profile of polydioxanone stents, in contrast to metallic stents which frequently induce early obstruction and mortality in this model [27].

Blood IL-8 levels were three orders of magnitude lower than those measured in tracheal lavage, indicating that the inflammatory response was predominantly local. A transient peak was observed on day 1 after implantation, which was significantly higher than baseline and subsequent measurements. Previous work using metallic stents in the same model showed that elevated blood IL-8 levels on day 1 predicted later tracheal stenosis [15]. In contrast, although polydioxanone stents induced a similar early IL-8 peak, no relevant late adverse effects were observed. This IL-8 peak was not observed in sham control animals, in which the surgical procedure was performed without stent deployment. Therefore, this early increase is more likely attributable to stent deployment rather than to the stent material itself.

Blood IL-8 levels in this study were lower than those reported for metallic stents by Arellano-Orden et al., whereas tracheal lavage IL-8 levels were higher [15]. These differences may reflect methodological variations in sample processing and IL-8 quantification, as well as a localized inflammatory response to polydioxanone degradation in the absence of the chronic mechanical and chemical stimuli induced by metallic stents.

The lack of statistically significant differences between the 30-, 60-, and 90-day groups may partly reflect limited statistical power; however, the strong and highly significant association between IL-8 levels and stent visibility suggests that degradation status, rather than time alone, may be a key biological factor influencing IL-8 expression.

Overall, clinical, endoscopic, and inflammatory findings consistently indicate that tracheal reactivity decreases over time, with a marked reduction after stent degradation. Similar reversibility of tissue injury after degradation has been reported in other studies of biodegradable airway stents [6,9,36].

This study has limitations. It was conducted in healthy rabbits, and although rabbit and human tracheas share important similarities, the findings cannot be fully extrapolated to pathological human airways. In addition, tracheoscopy was limited to the cranial trachea to avoid disturbing other parameters, which may have led to underestimation of distal lesions.

In conclusion, biodegradable polydioxanone tracheal stents are safe in this animal model and induce a moderate inflammatory response that largely resolves after stent degradation.

## 4. Materials and Methods

An experimental study was conducted in rabbits after approval by the Ethics Committee for Animal Experimentation of the University of Zaragoza (protocol PI25/17), in accordance with the ARRIVE guidelines. All procedures complied with Spanish Animal Protection Law RD 53/2013 and with European Union Directive 2010/63 on the protection of animals used for scientific purposes.

Twenty-one adult female New Zealand White rabbits (*Oryctolagus cuniculus*) weighing 4.62 ± 0.52 kg were included. Animals were randomly assigned to three groups (n = 7 per group) with predefined survival periods of 30, 60, or 90 days. In each group, six animals received a tracheal stent and one served as a sham control without stent implantation [36].

The stent was a self-expandable device made of 3.5-EP polydioxanone woven filament, with a gold radiopaque marker at each end (ELLA-CS s.r.o., Hradec Králové, Czech Republic). All stents measured 8 × 30 mm [36].

All procedures were performed under general anesthesia induced by intramuscular administration of medetomidine (0.5 mg/kg; Sedator^®^, Eurovet Animal Health, Bladel, The Netherlands) and ketamine (25 mg/kg; Imalgene 1000^®^, Merial, Sant Cugat del Vallès, Spain). A laryngeal mask airway was inserted to ensure adequate oxygenation and ventilation [36].

The ventral surface of the trachea was surgically exposed, and under fluoroscopic guidance the Seldinger technique was used to access the tracheal lumen and introduce the 11.8-F delivery system. Stents were deployed approximately 1 cm cranial to the carina. In sham control animals, the same procedure was performed using an empty delivery system. The tracheostomy was then sutured [36].

Clinical follow-up was performed throughout the study. Whole-blood samples for IL-8 quantification were collected before the procedure and on days 1, 3, 7, and 14, as well as at the end of the predefined survival period. After the final blood collection, tracheoscopy was performed and animals were euthanized by intravenous injection of sodium pentobarbital (120 mg/kg; Dolethal^®^, Vétoquinol, Madrid, Spain). Tracheal lavage was performed using 5 mL of sterile saline, and tracheal tissue samples were collected for histological analysis [36].

For IL-8 expression analysis, isolated leukocytes from blood samples and the cellular fraction of tracheal lavage were preserved in RNA stabilization solution (RNAlater™, Thermo Fisher Scientific, Vilnius, Lithuania) at −80 °C. RNA was extracted using the High Pure RNA Isolation Kit (Roche, Mannheim, Germany) at the Functional Genomics and Sequencing Unit of the Instituto Aragonés de Ciencias de la Salud (IACS).

Gene expression analysis was performed at the Support Service for Biomedical Research (SAIBIS) of the Instituto de Biomedicina de Sevilla (IBiS). Reverse transcription was carried out using TaqMan Gene Expression Assays (Thermo Fisher Scientific). IL-8 expression was quantified by real-time PCR using a ViiA 7 Real-Time PCR System (Thermo Fisher Scientific) with 96-well FAST plates. Prior to qPCR, cDNA concentrations were measured with a Nanodrop 2000C spectrophotometer and verified fluorometrically using a Qubit 3.0 platform (Thermo Fisher Scientific), and all samples were normalized to 5 ng/µL.

Four genes were analyzed: CXCL8 (IL-8) and the reference genes GAPDH, ACTB, and B2M, using the following probes: Oc03397860_m1 (CXCL8), Oc03823402_g1 (GAPDH), Oc03824857_g1 (ACTB), and Oc06779339_m1 (B2M) (Thermo Fisher Scientific). Relative gene expression was calculated using TaqMan technology.

Expression data were analyzed with SW Cloud (Thermo Fisher Scientific Inc., Waltham, MA, USA) and are reported as relative quantification (RQ) values, normalized to a reference (untreated) sample assigned an RQ value of 1.

**Figure 5:**
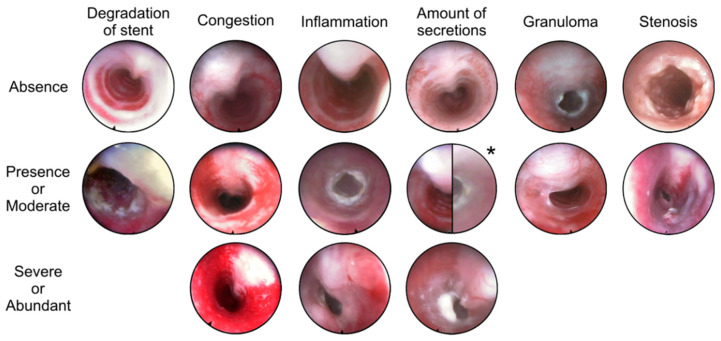
Rating of the parameters evaluated in tracheoscopies. * Moderate amount of secretions; right: in presence of a stent, left: without presence of a stent.

Tracheoscopic evaluation included assessment of stent degradation, congestion, inflammation, secretion accumulation, and the presence of granulomas or stenosis (Figure 5). Congestion refers to macroscopic vascular engorgement of the mucosa, whereas inflammation refers to visible epithelial changes such as edema, erythema, and friability; histological inflammation was evaluated separately based on cellular infiltrate and epithelial remodeling.

Statistical analyses were performed using SPSS Statistics for Macintosh (version 21.0; IBM Corp., Armonk, NY, USA). A significance level of 0.05 was used. Qualitative variables were compared using the likelihood-ratio test or Fisher’s exact test, as appropriate. Quantitative variables were analyzed after normality testing using Student’s *t*-test for independent samples or one-way analysis of variance (ANOVA) with Bonferroni post hoc correction. For non-normally distributed data, the Mann–Whitney U test or Kruskal–Wallis test was applied. Correlations were assessed using Pearson’s r or Spearman’s ρ, depending on data distribution.

## Figures and Tables

**Table 1:** Findings in Tracheoscopies in the Different Groups.

Findings		Group				*p* ^a^
		Control (*n* = 3)	30D (*n* = 6)	60D (*n* = 6)	90D (*n* = 6)	
Stent presence		-	100.0% *	50.0%	0.0% *	<0.001
Congestion	Absence	33.3%	33.3%	0.0%	0.0%	0.090
	Moderate	0.0%	16.7%	66.7%	66.7%	
	Severe	66.7%	50.0%	33.3%	33.3%	
Inflammation	Absence	100.0%	33.3% *	66.7%	100.0%	0.096
	Moderate	0.0%	50.0%	33.3%	0.0%	
	Severe	0.0%	16.7%	0.0%	0.0%	
Secretions	Absence	100.0% *	0.0%	0.0%	33.3%	0.007
	Moderate	0.0% *	83.3%	66.7%	66.7%	
	Abundant	0.0%	16.7%	33.3%	0.0%	
Granuloma		0.0%	0.0%	16.7%	16.7%	0.495
Stenosis		0.0%	16.7%	16.7%	0.0%	0.495

^a^ Significance according to likelihood-ratio test. * Observed value significantly lower or higher (*p* < 0.05) depending on the adjusted standardized residuals.

**Table 2:** Findings in Tracheoscopies According to Whether the Stent is Degraded.

Findings		Stent Present		*p* ^a^
		Yes (*n* = 9)	No (*n* = 9)	
Congestion	Absence	22.2%	0.0%	0.030 ^a^
	Moderate	22.2% *	77.8% *	
	Severe	55.6%	22.2%	
Inflammation	Absence	33.3% *	100.0% *	0.003 ^a^
	Moderate	55.6% *	0.0% *	
	Severe	11.1%	0.0%	
Secretions	Absence	0.0%	22.2%	0.030 ^a^
	Moderate	66.7%	77.8%	
	Abundant	33.3%	0.0%	
Granuloma		0.0%	22.2%	0.471 ^b^
Stenosis		11.1%	11.1%	1.000 ^b^

^a^ Significance according to likelihood-ratio test. ^b^ Significance according to Fisher’s exact test. * Observed value significantly lower or higher (*p* < 0.05) depending on the adjusted standardized residuals.

## Data Availability

The raw data supporting the conclusions of this article will be made available by the authors on request.

## Abbreviations

The following abbreviations are used in this manuscript:

IL-8	Interleukin 8
RQ	Relative Quantification
FDA	Food and Drug Administration
